# Nuclear factor-kappaB sensitizes to benzyl isothiocyanate-induced antiproliferation in p53-deficient colorectal cancer cells

**DOI:** 10.1038/cddis.2014.495

**Published:** 2014-11-20

**Authors:** N Abe, D-X Hou, S Munemasa, Y Murata, Y Nakamura

**Affiliations:** 1 Graduate School of Environmental and Life Science, Okayama University, Okayama, Japan; 2 Japan Society for the Promotion of Science, Tokyo, Japan; 3 Department of Biochemical Science and Technology, Faculty of Agriculture, Kagoshima University, Korimoto, Japan

## Abstract

Benzyl isothiocyanate (BITC), a dietary isothiocyanate derived from cruciferous vegetables, inhibits the proliferation of colorectal cancer cells, most of which overexpress *β*-catenin as a result of mutations in the genes for adenomatous polyposis coli or mutations in *β*-catenin itself. Because nuclear factor-*κ*B (NF-*κ*B) is a plausible target of BITC signaling in inflammatory cell models, we hypothesized that it is also involved in BITC-inhibited proliferation of colorectal cancer cells. siRNA-mediated knockdown of the NF-*κ*B p65 subunit significantly decreased the BITC sensitivity of human colorectal cancer HT-29 cells with mutated p53 tumor suppressor protein. Treating HT-29 cells with BITC induced the phosphorylation of I*κ*B kinase, I*κ*B-*α* and p65, the degradation of I*κ*B-*α*, the translocation of p65 to the nucleus and the upregulation of NF-*κ*B transcriptional activity. BITC also decreased *β*-catenin binding to a positive *cis* element of the cyclin D1 promoter and thus inhibited *β*-catenin-dependent cyclin D1 transcription, possibly through a direct interaction between p65 and *β*-catenin. siRNA-mediated knockdown of p65 confirmed that p65 negatively affects cyclin D1 expression. On the other hand, when human colorectal cancer HCT-116 cells with wild-type p53 were treated with BITC, translocation of p65 to the nucleus was inhibited rather than enhanced. p53 knockout increased the BITC sensitivity of HCT-116 cells in a p65-dependent manner, suggesting that p53 negatively regulates p65-dependent effects. Together, these results identify BITC as a novel type of antiproliferative agent that regulates the NF-*κ*B pathway in p53-deficient colorectal cancer cells.

Colorectal cancer is the third most common cancer in men and second in women worldwide.^1^ Colorectal cancer develops in a complex, multistep process involving progressive disruption of the homeostatic mechanisms that control epithelial proliferation, inflammation and differentiation. One of these disruptions is activation of the Wnt/*β*-catenin signaling pathway, which has an essential role in the tumorigenesis of colorectal cancer in human.^2^ In the absence of a Wnt signal, cytosolic *β*-catenin is normally bound to a *β*-catenin degradation complex, which causes it to be phosphorylated and then degraded by the ubiquitin–proteasome system. The *β*-catenin degradation complex is composed of adenomatous polyposis coli (APC), axin, casein kinase I*α* and glycogen synthase kinase 3. In response to the Wnt signal, *β*-catenin accumulates in the cytoplasm and is translocated to the nucleus, where it binds to T-cell factor (TCF)/lymphoid enhancer factor transcription factors and regulates the expression of target genes involved in the proliferation and invasiveness of cancer cells and angiogenesis.^3, 4^ Loss-of-function mutations in APC or mutations in *β*-catenin at the phosphorylation site, which are found in almost all human colorectal cancers, lead to stabilization of the *β*-catenin protein and aberrant activation of Wnt/*β*-catenin signaling. Accumulation of the overexpressed *β*-catenin in nucleus activates the expression of its target genes such as cyclin D1, which is required for the G_1_/S transition in the cell cycle.^5^ This transition contributes to cell proliferation and tumorigenesis in colorectal cancers.^6, 7, 8^ Thus, targeting the *β*-catenin/cyclin D1 pathway is a promising strategy for preventing the onset of colorectal cancer.

In addition to *β*-catenin, a transcriptional factor nuclear factor-*κ*B (NF-*κ*B) controls the proliferation of epithelial cells by regulating cyclin D1 expression.^9, 10^ NF-*κ*B regulates a wide variety of cellular genes involved in immunity, inflammation, cell proliferation and apoptosis. The p65 subunit of NF-*κ*B is a potent transcriptional activator and can also directly interact with DNA.^11^ NF-*κ*B in the cytosol of resting cells is bound to and inhibited by I*κ*B-*α* protein. Various stimuli including tumor necrosis factor-*α* and lipopolysaccharide enhance the phosphorylation of I*κ*B kinase (IKK), and then phosphorylated IKK (phospho-IKK) phosphorylates I*κ*B-*α* and p65. Phosphorylation of I*κ*B-*α* at Ser32/36 causes it to disassociate from NF-*κ*B, leading to its degradation by the ubiquitin–proteasome system.^12^ This allows NF-*κ*B to translocate to the nucleus where it regulates the expression of its target genes. Phosphorylation of p65 at Ser536 is also an indicator of NF-*κ*B activation.^13^ Cross talk between *β*-catenin and NF-*κ*B has a significant role in regulating the expression of their target genes.^14, 15, 16^ For example, p65 is recently reported to inhibit *β*-catenin binding on the positive *cis* element TCF-binding element0 (TBE0) site of the cyclin D1 promoter and thus its transcription.^17^ Therefore, NF-*κ*B has attracted much attention as a novel regulator of the *β*-catenin/cyclin D1 pathway in colorectal cancer cells.

Isothiocyanates (ITCs), mainly derived from cruciferous vegetables such as broccoli, wasabi (Japanese horseradish) and watercress, are highly effective in chemoprevention and have antitumor activities *in vitro* and *in vivo*.^18^ Dietary consumption of ITC-containing foods has been inversely related to the risk of colorectal cancer in human.^19^ We previously demonstrated that benzyl ITC (BITC), an ingredient in papaya,^20^ inhibits cell proliferation by inducing cell cycle arrest and apoptosis through the mitogen-activated protein kinase pathways in human T-lymphocytic leukemia Jurkat cells.^21^ In human colorectal cancer cells, BITC also inhibits cell proliferation by stimulating apoptosis.^22^ By regulating NF-*κ*B, BITC reduces inflammation in RAW264.7 murine macrophages and reduces migration of MDA-MB-231 human breast cancer cells.^23, 24^ Although BITC might target NF-*κ*B, it is unclear whether such targeting regulates colorectal cancer cell proliferation.

In the present study, we investigated the role of NF-*κ*B in BITC-inhibited colorectal cancer cell proliferation. Here we demonstrate that NF-*κ*B sensitizes to BITC-induced antiproliferation in human colorectal cancer HT-29 cells. Our results indicate that BITC inhibits *β*-catenin-dependent cyclin D1 transcription and cell proliferation by causing p65 to accumulate in the nucleus. Furthermore, experiments with two other colorectal cancer cell lines (HCT-116 p53^+/+^ and HCT-116 p53^−^^/−^) revealed that p53 tumor suppressor protein negatively regulates the effects of p65 in BITC signaling. This study provides the evidence showing that NF-*κ*B represents a novel therapeutic target of the ITC-based prevention of colorectal cancer with p53 mutation and *β*-catenin overexpression.

## Results

### NF-*κ*B sensitizes to antiproliferation by BITC in HT-29 cells

**Figure 1:**
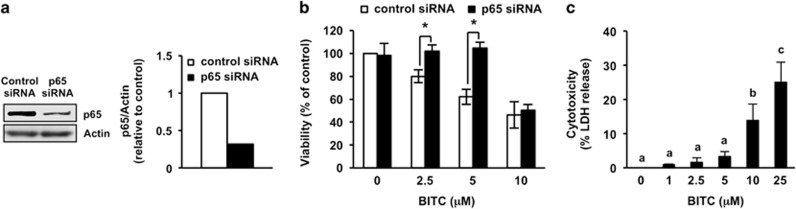
Effects of p65 knockdown on antiproliferation by BITC in HT-29 cells. (**a**) Knockdown of p65 by RNAi in HT-29 cells. HT-29 cells were transfected with control siRNA or p65 siRNA. Whole-cell lysates were prepared and western blot analysis was performed for p65 and actin. (**b**) Effects of p65 knockdown on antiproliferation by BITC in HT-29 cells. HT-29 cells were transfected with control siRNA or p65 siRNA and exposed to the indicated concentrations of BITC for 24 h and cell viability was determined by trypan blue dye exclusion assay. The values represent means±S.D. of three separate experiments (*P<0.05 compared between the indicated groups; Student's *t*-test). (**c**) Cytotoxicity of BITC to HT-29 cells. HT-29 cells were exposed to the indicated concentrations of BITC for 24 h and cytotoxicity was determined by LDH release assay. Data were analyzed by a one-way analysis of variance (ANOVA) followed by multiple comparisons among means (Tukey's HSD) using XLSTAT software (Addinsoft, Paris, France). Different letters above the bars indicate significant differences among treatments for each compound (*P*<0.05)

The HT-29 cell line is commonly used as a colorectal cancer model because it has loss-of-function mutations in APC.^25^ An immunoblot analysis showed that the transfection of HT-29 cells with p65-specific siRNA depleted the p65 level by 68% compared with control (Figure 1a). BITC dose-dependently suppressed the viability of HT-29 cells transfected with control siRNA, whereas siRNA-mediated knockdown of p65 significantly counteracted the antiproliferation induced by 2.5 and 5 *μ*M BITC but not by 10 *μ*M BITC (Figure 1b). Lactate dehydrogenase (LDH) release that is used as an index of cytotoxicity was drastically increased by the treatment of >10 *μ*M BITC (Figure 1c). These results indicate that NF-*κ*B has a significant role in cell growth inhibition, rather than cell death by BITC in human colorectal cancer cells.

### BITC activates NF-*κ*B signaling pathway in HT-29 cells

**Figure 2:**
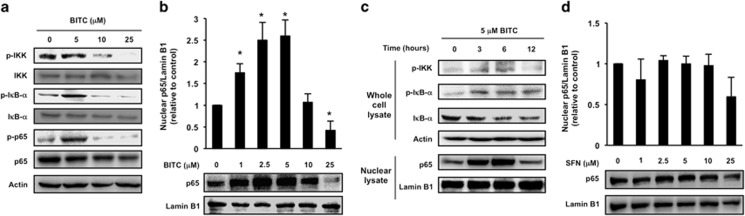
Effects of BITC and SFN on the expressions of NF-*κ*B signaling proteins in HT-29 cells. HT-29 cells were treated with BITC or SFN and subjected to western blot analysis. (**a**) Whole-cell lysates, p-IKK, IKK, p-I*κ*B-*α*, I*κ*B-*α*, p-p65, p65 and actin. Three-hour BITC treatment. (**b**) Nuclear lysates, p65 and lamin B1. Six-hour BITC treatment. (**c**) Whole-cell lysates (upper panel) and nuclear lysates (lower panel), p-IKK, p-I*κ*B-*α*, I*κ*B-*α*, actin, p65 and lamin B1. (**d**) Nuclear lysates, p65 and lamin B1. Six-hour SFN treatment. The values represent means±S.D. of three separate experiments (*P<0.05 compared with control; Student's *t*-test)

As shown in Figure 2a, 5 *μ*M BITC increased the phosphorylation of IKK, I*κ*B-*α* and p65 (at Ser176/180, Ser32/36 and Ser536, respectively), whereas a higher concentration of BITC (25 *μ*M) decreased the phosphorylation of these residues. Consistently, 1–5 *μ*M BITC significantly increased, but 25 *μ*M BITC decreased, the nuclear translocation of p65 (Figure 2b). The increases of phospho-IKK and phospho-I*κ*B-*α* and the decrease of total I*κ*B-*α* were time dependent (Figure 2c). The increase in nuclear p65 was apparent 3 h post treatment of BITC and peaked at 6 h. In contrast, sulforaphane (SFN), a naturally occurring aliphatic ITC in broccoli, at concentrations from 1–25 *μ*M, did not increase the nuclear p65 level (Figure 2d). These results suggest that not all ITCs are activators of NF-*κ*B signaling in HT-29 cells.

### BITC modulates cyclin D1 transcription by modifying the promoter binding of *β*-catenin

**Figure 3:**
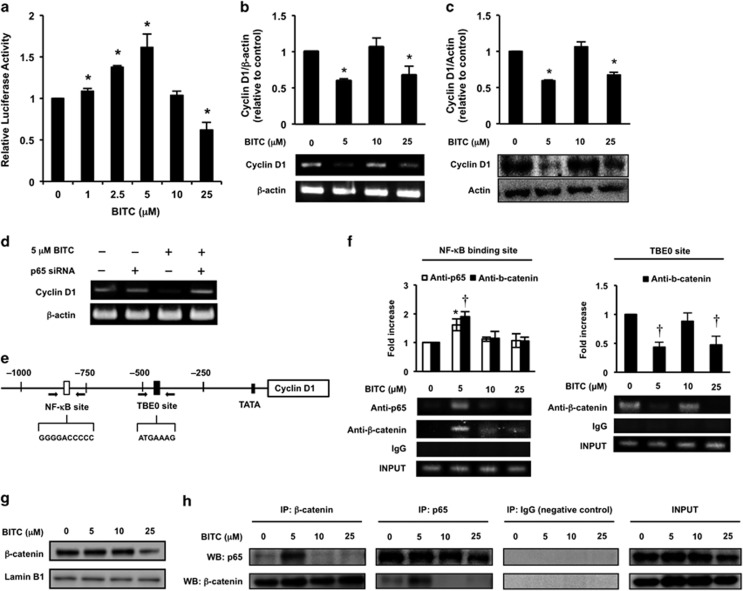
Involvement of NF-*κ*B in the regulation of *β*-catenin-dependent cyclin D1 expression by BITC. (**a**) Effects of BITC on transcriptional activity of NF-*κ*B in HT-29 cells. Cells were cotransfected with 1 *μ*g of pNF-*κ*B-Luc and 1 *μ*g of pRL-TK vector for 48 h and treated with BITC for 6 h. After treatment, cells were analyzed using a Dual-Luciferase Reporter Assay System. The values represent means±S.D. of three separate experiments (*P<0.05 compared with control; Student's *t*-test). (**b**) Effects of BITC on the gene expression of cyclin D1. HT-29 cells were treated with BITC for 6 h. The mRNA levels of cyclin D1 and *β*-actin were determined by RT-PCR. The values represent means±S.D. of three separate experiments (*P<0.05 compared with control; Student's *t*-test). (**c**) Effects of BITC on the protein expression of cyclin D1. HT-29 cells were treated with BITC for 24 h. Whole-cell lysates were prepared and western blot analysis was performed for cyclin D1 and actin. The values represent means±S.D. of three separate experiments (*P<0.05 compared with control; Student's *t*-test). (**d**) Effects of p65 knockdown on BITC-decreased cyclin D1 gene expression. HT-29 cells were transfected with control siRNA or p65 siRNA and exposed to 5 *μ*M BITC for 6 h. The mRNA expression of cyclin D1 and *β*-actin were determined using RT-PCR. (**e**) Schematic representation of the human cyclin D1 promoter. NF-*κ*B binding site, TBE0 binding site and their sequences are shown. Small arrows indicate the positions and directions of the PCR primers used for the ChIP assay. (**f**) Binding of *β*-catenin or p65 to the NF-*κ*B binding site and the TBE0 site on cyclin D1 promoter. After treatment with BITC for 6 h, HT-29 cells were cross-linked with 1% of formaldehyde for ChIP assay. Chromatin fragments were immunoprecipitated with antibodies against *β*-catenin, p65 or goat IgG (negative control) and the cyclin D1 promoter regions were amplified by PCR. The values represent means±S.D. of three separate experiments (*P<0.05 compared with control of anti-p65 group and ^†^*P*<0.05 compared with control of anti-*β*-catenin group; Student's *t*-test). (**g**) Effects of BITC on nuclear *β*-catenin level in HT-29 cells. HT-29 cells were treated with different concentrations of BITC for 6 h. Nuclear lysates were prepared and western blot analysis was performed for *β*-catenin and lamin B1. (**h**) Detection of the interaction between *β*-catenin and p65 using immunoprecipitation assay. HT-29 cells were treated with BITC for 6 h and immunoprecipitated with antibodies against *β*-catenin, p65 or goat IgG (negative control). Western blot analysis was performed for p65 and *β*-catenin

**Figure 4:**
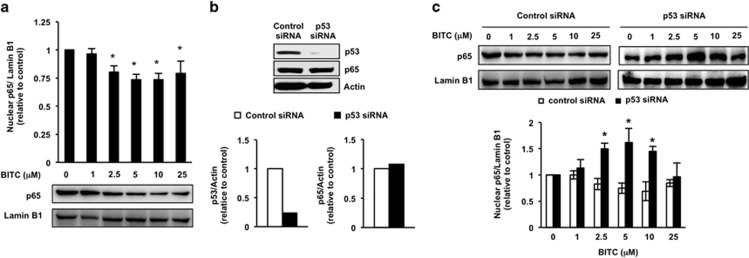
Effects of p53 knockdown on nuclear translocation of p65 by BITC in HCT-116 p53^+/+^ cells. (**a**) Effects of BITC on nuclear p65 level in HCT-116 p53^+/+^ cells. HCT-116 p53^+/+^ cells were treated with the indicated concentrations of BITC for 6 h. Western blot analysis of nuclear lysates was performed for p65 and lamin B1. The values represent means±S.D. of three separate experiments (*P<0.05 compared with control; Student's *t*-test). (**b**) Knockdown of p53 by RNAi. HCT-116 p53^+/+^ cells were transfected with control siRNA or p53 siRNA. Whole-cell lysates were prepared and western blot analysis was performed for p53, p65 and actin. (**c**) Effects of BITC on nuclear p65 level in p53 siRNA-transfected HCT-116 p53^+/+^ cells. HCT-116 p53^+/+^ cells were transfected with control siRNA or p53 siRNA and treated with the indicated concentrations of BITC for 6 h. Western blot analysis of nuclear lysates was performed for p65 and lamin B1. The values represent means±S.D. of three separate experiments (*P<0.05 compared with control of p53 siRNA group; Student's *t*-test)

**Figure 5:**
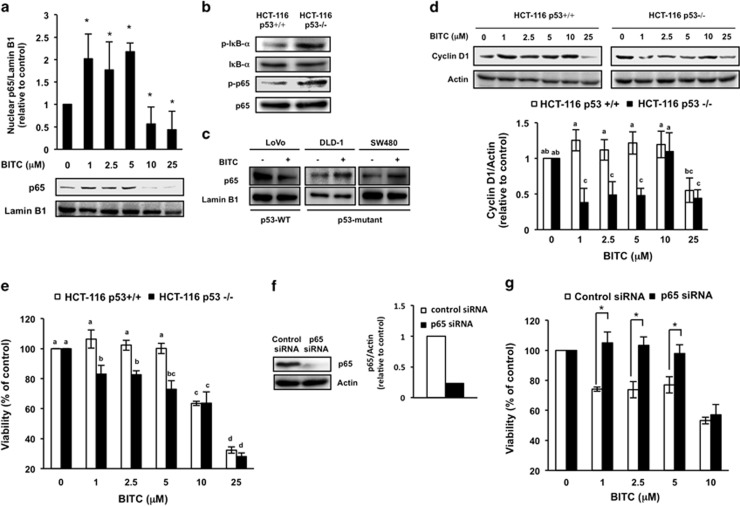
Effects of p53 deficiency on antiproliferation by BITC in colorectal cancer cells. (**a**) Effects of BITC on nuclear p65 level in HCT-116 p53^−/−^ cells. HCT-116 p53^−/−^ cells were treated with the indicated concentrations of BITC for 6 h. Western blot analysis of nuclear lysates was performed for p65 and lamin B1. The values represent means±S.D. of three separate experiments (*P<0.05 compared with control; Student's *t*-test). (**b**) The basal phosphorylation levels of I*κ*B-*α* at Ser32/36 and p65 at Ser536 in HCT-116 p53^+/+^ and HCT-116 p53^−/−^ cells. Whole-cell lysates of HCT-116 p53^+/+^ and HCT-116 p53^−/−^ cells were prepared and western blot analysis was performed for phospho-I*κ*B-*α* (Ser32/36), I*κ*B-*α*, phospho-p65 (Ser536) and p65. (**c**) Effects of BITC on nuclear p65 level in other colorectal cancer cells. Indicated colorectal cancer cell lines with different p53 statuses were treated with 2.5 *μ*M BITC for 6 h. Western blot analysis of nuclear lysates was performed for p65 and lamin B1. (**d**) Effects of BITC on the protein expression of cyclin D1 in HCT-116 p53^+/+^ and HCT-116 p53^−/−^ cells. Both cells were treated with BITC for 24 h. Whole-cell lysates were prepared and western blot analysis was performed for cyclin D1 and actin. The values represent means±S.D. of three separate experiments. Data were analyzed by a one-way ANOVA followed by Tukey's HSD using XLSTAT software. Different letters above the bars indicate significant differences among treatments for each compound (*P*<0.05). (**e**) Effects of BITC on cell viability in HCT-116 p53^+/+^ and HCT-116 p53^−/−^ cells. Both cells were exposed to different concentrations of BITC for 24 h and cell viability was determined by MTT assay. Data were analyzed by a one-way ANOVA followed by Tukey's HSD using XLSTAT software. Different letters above the bars indicate significant differences among treatments for each compound (*P*<0.05). (**f**) Knockdown of p65 by RNAi in HCT-116 p53^−/−^ cells. HCT-116 p53^−/−^ cells were transfected with control siRNA or p65 siRNA. Whole-cell lysates were prepared and western blot analysis was performed for p65 and actin. (**g**) Effects of p65 knockdown on antiproliferation by BITC in HCT-116 p53^−/−^ cells. HCT-116 p53^−/−^ cells were transfected with control siRNA or p65 siRNA and exposed to the indicated concentrations of BITC for 24 h and cell viability was determined by trypan blue dye exclusion assay. The values represent means±S.D. of three separate experiments (*P<0.05 compared between the indicated groups; Student's *t*-test)

Consistent with the effects of BITC on NF-*κ*B signaling pathway (Figures 2a and b), 1–5 *μ*M BITC significantly increased the transcriptional activity of NF-*κ*B, whereas 25 *μ*M BITC decreased it (Figure 3a). At both the mRNA and protein levels, cyclin D1 was decreased by the treatment of BITC at 5 and 25 *μ*M (Figures 3b and c). Moreover, siRNA-mediated knockdown of p65 canceled the suppression of cyclin D1 gene expression by 5 *μ*M BITC (Figure 3d). We also examined the effects of BITC on mRNA levels of the NF-*κ*B-targeted genes other than cyclin D1. The tendency of the dose-dependent effect of BITC on c-myc was quite similar to that on cyclin D1, even though the expression of interferon-*γ* (IFN-*γ*) was enhanced by 2.5 *μ*M BITC (Supplementary Figure 1). The binding of *β*-catenin and p65 on the NF-*κ*B binding site was enhanced by treatment of 5 *μ*M BITC and the *β*-catenin binding on the TBE0 site was suppressed by treatment of 5 and 25 *μ*M BITC (Figure 3f). In addition, 25 *μ*M BITC decreased the nuclear *β*-catenin level in HT-29 cells (Figure 3g). Treatment of 5 *μ*M BITC enhanced the interaction between *β*-catenin and p65 (Figure 3h). These results suggest that the interference of *β*-catenin binding on the TBE0 site by p65 is involved in the suppression of cyclin D1 gene expression by BITC.

### p53 negatively regulates BITC-activated NF-*κ*B signaling pathway

HCT-116 p53^+/+^ cells are often used as a colorectal cancer model because they overexpress *β*-catenin.^26^ In contrast to HT-29 cells, HCT-116 p53^+/+^ cells show a decreased nuclear translocation of p65 in the presence of 2.5–25 *μ*M BITC (Figure 4a), accompanied by a decreased phosphorylation of I*κ*B-*α* at Ser32/36 (Supplementary Figure 2). As the p53 status is one of the differences between these cell lines, we hypothesized that p53 inhibits the BITC-activated NF-*κ*B signaling pathway in HCT-116 p53^+/+^ cells. To test this hypothesis, we examined whether p53 knockdown changes the effect of BITC on the nuclear level of p65 in HCT-116 p53^+/+^ cells. Immunoblot analysis indicated that transfection of p53-specific siRNA depleted the p53 level by 80% and did not affect the p65 level (Figure 4b). As shown in Figure 4c, 2.5–10 *μ*M BITC significantly enhanced the nuclear translocation of p65 in p53 siRNA-treated HCT-116 p53^+/+^ cells but not in control siRNA-treated cells. We further confirmed the dependency of BITC-activated NF-*κ*B signaling on p53 status by using a p53 knockout cell line, HCT-116 p53^−/−^. As shown in Figure 5a, 1–5 *μ*M BITC significantly enhanced the nuclear translocation of p65 in HCT-116 p53^−/−^ cells. We also found that the basal phosphorylation levels of I*κ*B-*α* at Ser32/36 and p65 at Ser536 in HCT-116 p53^−/−^ cells were high compared with those in HCT-116 p53^+/+^ cells (Figure 5b). The negative regulating role of p53 in the NF-*κ*B translocation was also confirmed in other colorectal cancer cells including LoVo cells (p53 wild type) and, DLD-1 and SW480 cells (p53 mutated) (Figure 5c).

To confirm the idea that p53 negatively regulates the antiproliferation by BITC in colorectal cancer cells, we compared the effects of BITC on cyclin D1 expression and cell viability in HCT-116 p53^+/+^ and HCT-116 p53^−/−^ cells. BITC (1–5 *μ*M) significantly decreased cyclin D1 expression in HCT-116 p53^−/−^ cells (Figure 5d) and significantly decreased their viability (Figure 5e) relative to HCT-116 p53^+/+^ cells. At BITC concentrations >10 *μ*M, the decreases of cyclin D1 expression and cell viability were not significantly different between HCT-116 p53^+/+^ and HCT-116 p53^−/−^ cells. Moreover, 77% knockdown of p65 in HCT-116 p53^−/−^ cells (see Figure 5f) significantly counteracted the antiproliferation induced by 1–5 *μ*M BITC but not the antiproliferation induced by 10 *μ*M BITC (Figure 5g). These results strongly suggest that p53 negatively regulates NF-*κ*B-dependent antiproliferation by BITC in colorectal cancer cells.

## Discussion

The present results demonstrate that NF-*κ*B sensitizes to BITC-induced antiproliferation in p53-deficient human colorectal cancer cells. Knockdown of p65 decreased the BITC sensitivity of HT-29 (Figure 1b) and HCT-116 p53^−/−^ cells (Figure 5g). BITC significantly induced the nuclear translocation of the NF-*κ*B p65 subunit in HT-29 (Figure 2b), HCT-116 p53^−/−^ (Figure 5a), DLD-1 and SW480 cells (Figure 5c). NF-*κ*B-targeting anticancer agents include nonsteroidal antiinflammatory drugs (NSAIDs), such as aspirin. Aspirin has been reported to inhibit the proliferation of colorectal cancer cells by inhibiting NF-*κ*B transcriptional activity.^27^ BITC is a food-derived compound that is known to regulate the proliferation of colorectal cancer cells. To our knowledge, this is the first report that shows its antiproliferation activity is sensitized by NF-*κ*B. In human lung cancer A549 cells, both BITC and SFN exert their anticancer effects through binding to tubulin.^28^ However, in HT-29 cells, the nuclear translocation of p65 was induced by BITC (Figure 2b) but not by SFN (Figure 2d). BITC has a higher hydrophobicity than SFN owing to its aromatic ring,^29^ which may cause it to target molecules other than tubulin. Although further study is needed to clarify the underlying mechanism, it is noteworthy that regulation of NF-*κ*B depends on the structure of the ITC.

Although numerous studies have established NF-*κ*B as a tumor-promoting transcription factor,^30^ recent studies have shown that NF-*κ*B can also act as a tumor suppressor.^31, 32, 33, 34^ Because NF-*κ*B activity enhances sensitivity to cytotoxic chemotherapy in certain cancer cell lines, the two opposing roles of NF-*κ*B may be explained by the other oncogenic status.^35^ For example, as *β*-catenin is overexpressed in colorectal cancers and functions as an oncogene, NF-*κ*B could act as a tumor suppressor in colorectal cancer cells. Low concentrations of BITC (1–5 *μ*M) increased the transcriptional activity of NF-*κ*B (Figure 3a), whereas they decreased or did not affect the expression of genes whose promoters contain binding sites for *β*-catenin/TCF and NF-*κ*B: cyclin D1^5, 9, 10^ (Figure 3b); c-myc;^36, 37^ or COX-2^38, 39^ (Supplementary Figure 1). To the best of our knowledge, the IFN-*γ* promoter contains the binding sites of NF-*κ*B but not that of *β*-catenin/TCF.^40^ The mRNA level of IFN-*γ* was increased by a low concentration of BITC (2.5 *μ*M; Supplementary Figure 1). This is consistent with previous findings that cross talk between *β*-catenin/TCF and NF-*κ*B has a pivotal role in regulating the expression of their targeted genes.^14, 15, 16^ Furthermore, p65-dependent effects of BITC are not observed in p53-positive cancer cells (Figures 4a,5d and e) and thus are presumably not observed in normal tissue. As the possibility that BITC-induced NF-*κ*B activation leads to invasion and metastasis of p53-deficient cancer cells could not be excluded in this study, further studies are needed to check the side effects of NF-*κ*B activation.

Inhibiting the *β*-catenin/cyclin D1 pathway can prevent the onset of colorectal cancer. Little is known about the antiproliferating mechanism of the *β*-catenin targeting anticancer agents other than that it involves reducing the *β*-catenin level^41^ and attenuating the transcriptional activity of *β*-catenin/TCF complex.^42^ p65 was recently shown to repress *β*-catenin-dependent cyclin D1 transcription, possibly through a protein–protein interaction.^43^ This model proposes that mRNA expression of cyclin D1 is negatively regulated by p65 through the interference of *β*-catenin binding on the TBE0 site. Consistent with this repression model of p65, 5 *μ*M BITC enhanced the interaction between *β*-catenin and p65 (Figure 3h), inhibited the *β*-catenin binding to the TBE0 site on the cyclin D1 promoter (Figure 3f) and decreased cyclin D1 expression (Figures 3b and c). On the other hand, at a concentration of 25 *μ*M, BITC decreased cyclin D1 expression independently of p65 (Figures 3b and c) possibly by decreasing the binding of *β*-catenin to the TBE0 site by downregulating the expression of nuclear *β*-catenin (Figures 3f and g). An epidemiological study suggested that patients with a combination of cyclin D1 A870G polymorphism, low dietary ITC consumption and high-activity glutathione *S*-transferase profile have an increased risk of colorectal cancer.^44^ In colorectal cancer model Apc (Min/+) mice, phenethyl ITC, a dietary ITC with an aromatic ring like BITC, decreased cyclin D1 expression and polyposis formation but not *β*-catenin total expression.^45^ These findings strongly support the idea that dietary aromatic ITCs elicit chemopreventive effects, possibly by regulating cyclin D1 expression in colorectal cancers.

Our data indicate that p53 weakens the inhibition of cyclin D1 expression and cell proliferation by BITC by blocking the activation of NF-*κ*B signaling pathway in colorectal cancer cells. HCT-116 p53^+/+^ cells have a wild-type p53, whereas the p53 of HT-29 cells has a loss-of-function mutation at codon 273.^46^ The present results show that BITC increases the levels of phospho-I*κ*B-*α* and nuclear p65 in (p53 mutated) HT-29 cells (Figures 2a and b), whereas both are decreased in HCT-116 p53^+/+^ cells (Figure 4a and Supplementary Figure 2). We also found that in HCT-116 cells with p53 knockout, BITC increased nuclear translocation of p65 (Figure 5a) and decreased cyclin D1 expression and cell viability (Figures 5d and e). Tumor suppressor protein p53 has a key role in cellular responses to DNA damage. p53 inactivates NF-*κ*B signaling by reducing the catalytic activity of IKK through the inhibition of *O*-linked *β*-*N*-acetyl glucosamine (*O*-GlcNAc) modification.^47^ Thus, p53 might block BITC-activated NF-*κ*B signaling pathway by inhibiting *O*-GlcNAc modification of IKK. This idea is also supported by the observation that the basal phosphorylation levels of I*κ*B-*α* at Ser32/36 and p65 at Ser536 in HCT-116 p53^−/−^ cells were much higher than those in HCT-116 p53^+/+^ cells (Figure 5b). We previously reported that p53 negatively regulates the cytotoxicity by BITC in normal colorectal CCD-18Co cells.^48^ Consistent with this report, we showed in Figure 5e that HCT-116 p53^+/+^ cells are more resistant to antiproliferation by BITC than HCT-116 p53^−/−^ cells. BITC might decrease phospho-I*κ*B-*α* level and nuclear p65 level through the decrease of p-IKK catalytic activity by increasing p53 level in p53-positive cells. Taken together, these findings suggest that p53 is a negative regulator of antiproliferation of colorectal cancer cells by BITC. In addition, BITC did neither significantly affect cyclin D1 expression in HCT-116 p53^+/+^ cells, nor significantly increase their viability, although further studies are needed to check whether BITC increases cancer risk in the other p53-positive cell lines and tissues.

Our results also indicate that the antiproliferation effects of BITC depend on its concentration. NF-*κ*B-dependent antiproliferation effects of BITC were only observed at the limited concentrations (1–5 *μ*M). More than 10 *μ*M of BITC drastically increased cell death in HT-29 cells (Figure 1c) without NF-*κ*B dependency on antiproliferation effect (Figure 1b). In a preliminary study, we also found that 10 and 25 *μ*M of BITC-induced apoptotic cell death but not necrosis (unpublished data). These results suggest that low concentrations of BITC mainly induce cell growth inhibition through an NF-*κ*B-dependent pathway only in p53-deficient cells, whereas higher concentrations of BITC induce apoptotic cell death with the decrease of nuclear NF-*κ*B in both p53-positive and -deficient cells. Such dose-specific cellular responses to BITC were also observed in human T-lymphocytic leukemia Jurkat cells; BITC induced the activation of c-Jun N-terminal kinase at 5 *μ*M, but not at >25 *μ*M^49^ and induced apoptosis at low concentration but induced necrosis at high concentration.^50^ The electrophilic moiety of BITC is reported to covalently bind to cysteine and lysine residues *in vitro*^51^ and to cysteine residues *in vivo*^28^. The number of species of cellular proteins that are modified by ITCs increases with their increasing concentration.^52^ Therefore, high doses of BITC may nonspecifically target various proteins to inhibit cell proliferation independently of NF-*κ*B signaling.

**Figure 6:**
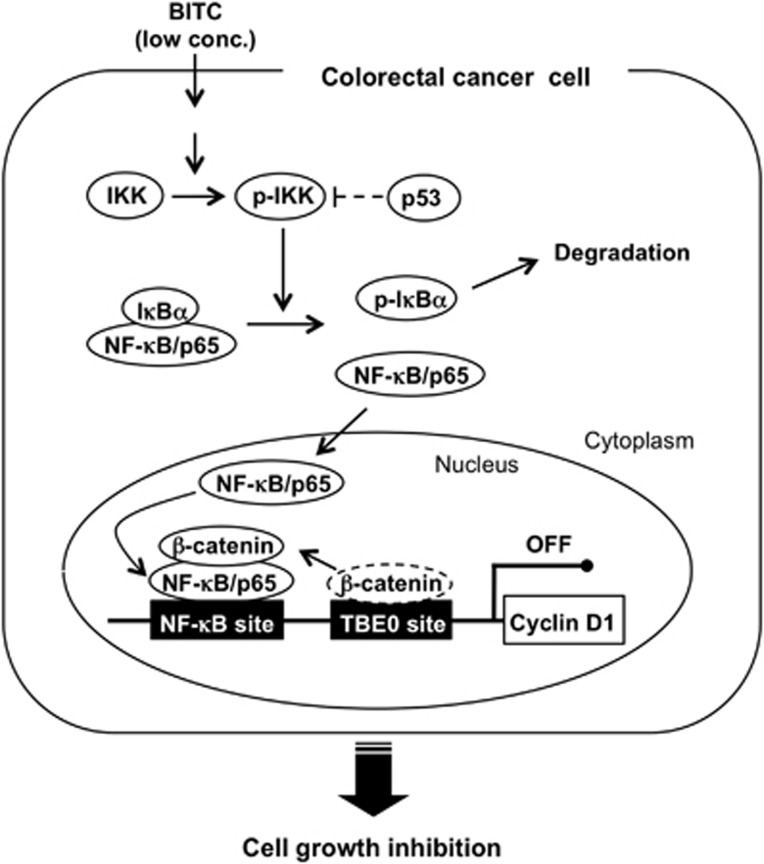
Proposed mechanism for the antiproliferation by BITC. BITC activates NF-*κ*B signaling pathway. p53 inhibits catalytic activity of IKK*β*.^47^ Lower concentrations of BITC enhance the interaction between p65 and *β*-catenin, which blocks the binding of *β*-catenin to the TBE0 site (a positive *cis* element of the cyclin D1 promoter). BITC then inhibits cyclin D1 expression and cell growth in colorectal cancer cells

In conclusion, we have demonstrated that NF-*κ*B sensitizes to BITC-induced antiproliferation in p53-deficient colorectal cancer cells. BITC enhances the interaction between p65 and *β*-catenin to block the *β*-catenin binding to the positive *cis* element of the cyclin D1 promoter and then inhibits cyclin D1 expression and cell proliferation. Furthermore, p53 blocks BITC-induced nuclear translocation of p65 and downregulates BITC-inhibited cyclin D1 expression and cell proliferation. Taken together, our results suggest that BITC inhibits *β*-catenin-dependent cyclin D1 transcription and cell proliferation through the nuclear translocation of p65 in human colorectal cancer cells (Figure 6). Thus, we identify NF-*κ*B as a novel therapeutic target in p53-deficient colorectal cancer cells, which contributes to our understanding of the complex intracellular signaling cascades that regulate cell proliferation. After consumption of cruciferous vegetables, plasma concentrations of ITC metabolites peak at a few *μ*mol/l.^53, 54^ However, a recent preclinical evaluation revealed that the concentrations of ITCs in the gastric lumina temporally reached 600–2000 *μ*M after the consumption of broccoli extract.^55^ Therefore, the concentrations of BITC used in this study are locally achievable at the colorectum, but it is unclear whether ITC metabolites such as ITC-glutathione conjugates have antiproliferative effects. Further studies are needed to determine the *in vivo* effects of ingested ITCs on colorectal cancer cells, as well as the primary target to activate the NF-*κ*B pathway by BITC.

## Materials and Methods

### Chemicals and antibodies

BITC and SFN were purchased from LKT Laboratories, Inc. (St. Paul, MN, USA). Antibodies against phosphorylated NF-*κ*B p65 (phospho-NF-*κ*B p65 and Ser536), phospho-IKK*α/β* (Ser176/180), phosphorylated I*κ*B-*α* (phospho-I*κ*B-*α* and Ser32/36) and IKK were purchased from Cell Signaling Technology, Inc. (Beverly, MA, USA). Protein A/G PLUS-Agarose Immunoprecipitation reagent, siRNAs for NF-*κ*B p65 and p53, control siRNA, siRNA transfection medium, siRNA transfection reagent, antibodies against NF-*κ*B p65, I*κ*B-*α*, lamin B1, actin, *β*-catenin and p53 and horseradish peroxidase-linked antirabbit and antimouse IgGs were purchased from Santa Cruz Biotechnology (Santa Cruz, CA, USA). Protease inhibitor cocktail was purchased from Sigma-Aldrich (St. Louis, MO, USA). McCoy's 5A, RPMI1640, Leibovitz's L15 and HamF12 medium, penicillin/streptomycin, Trypan blue stain, Lipofectamine 3000 and Trizol reagent were purchased from Life technologies (Carlsbad, CA, USA). pNF-*κ*B-Luc was purchased from Agilent Technologies, Inc. (Santa Clara, CA, USA). pRL-TK vector and Dual-Luciferase Reporter Assay System were purchased from Promega (Madison, WI, USA). Fatal bovine serum (FBS) was purchased from Nichirei Corporation (Tokyo, Japan). Bio-Rad Protein Assay was purchased from Bio-Rad Laboratories (Hercules, CA, USA). Chemi-Lumi One Super was purchased from Nakalai Tesque Inc. (Kyoto, Japan). Immobilon-P membrane was purchased from Merck Millipore (Billerica, MA, USA). M-MLV reverse transcriptase and Taq polymerase were purchased from Takara Bio Inc. (Shiga, Japan). Salmon sperm DNA was purchased from BioDynamics Laboratory (Tokyo, Japan). All other chemicals were purchased from Wako Pure Chemical Industries (Osaka, Japan).

### Human colorectal cancer cell lines

HT-29 cells and HCT-116 p53^+/+^ cells were obtained from the American Type Culture Collection (Manassas, VA, USA). HCT-116 p53^−/−^ cells were kindly provided by Dr. Bert Vogelstein (Johns Hopkins Medical Institute, Baltimore, MD, USA). DLD-1 cells and SW480 cells were obtained from Tohoku University Cell Resource Center for Biomedical Research (Miyagi, Japan). LoVo cells were obtained from RIKEN BioResource Center Cell Bank (Ibaraki, Japan). HT-29, HCT-116 p53^+/+^ and HCT-116 p53^−/−^ cells were maintained in McCoy's 5A medium. DLD-1, SW480 and LoVo cells were maintained in RPMI1640, Leibovitz's L15 and HamF12 medium, respectively. All media were supplemented with 10% heat-inactivated FBS and 1% penicillin/streptomycin. Cells were grown at 37 °C in an atmosphere of 95% O_2_ and 5% CO_2_. Confluent cells were exposed to the test compounds (resolved in 0.2% DMSO) in the medium containing 0.5% FBS.

### RNA interference

Cells were cultured in six-well plates (2 × 10^5^ cells/well) in normal growth medium without antibiotic and transfected with siRNA. Predesigned siRNAs targeting p65 and p53 or nonspecific control siRNAs were transfected to the cells according to the manufacturer's instructions using siRNA transfection medium and siRNA transfection reagent. After 72-h incubation, cells were assayed using the appropriate protocol.

### Trypan blue dye exclusion assay

Trypan blue dye exclusion assay was carried out for quantitative analysis of cell viability. Cell suspensions were mixed with 0.4% Trypan blue stain. The total cells and viable cells (cells that excluded blue dye) were counted using a hemocytometer (Bürker-Türk, Hirschmann Laborgeräte GmbH & Co. KG, Eberstadt, Germany) under a light microscope.

### LDH-release assay

LDH-release assay was carried out for the quantitative determination of cytotoxicity. Cells were seeded in 96-well plates at a density of 2 × 10^4^ cells/well in culture medium. After incubation, cells were treated with BITC for 24 h. LDH activity was measured by using an LDH-Cytotoxicity Test Wako, in accordance with the manufacturer's instructions. The absorbance was measured at 560 nm. Total LDH release (100%) was obtained by the treatment of 0.1% Tween 20.

### Western blot analysis

Cells were washed with ice-cold phosphate-buffered saline without calcium and magnesium (PBS (-)). Whole-cell lysates were prepared in lysis buffer (20 mM Tris-HCl pH 7.5, 150 mM NaCl, 2 mM EDTA, 2 mM EGTA, 2 mM DTT, 10 mM NaF, 1 mM Na_3_VO_4_, 1 mM PMSF, 1% SDS and 1% Triton-X-100) containing protease inhibitor cocktail and left on ice for 20 min. After sonication, lysates were centrifuged and the supernatant was used as whole-cell lysates. For preparation of nuclear lysates, cells were suspended with buffer-1 (10 mM HEPES pH 7.9, 10 mM KCl, 0.1 mM EDTA, 0.1 mM EGTA, 1 mM DTT, 10 mM NaF, 1 mM Na_3_VO_4_ and 1 mM PMSF) containing protease inhibitor cocktail and left on ice for 15 min. After addition with 0.4% NP-40, the mixture was centrifuged at 500 × g for 4 min. Pellets were washed with buffer-1 for three times and suspended with buffer-2 (20 mM HEPES pH 7.9, 400 mM NaCl, 1 mM EDTA, 1 mM EGTA, 1 mM DTT, 10 mM NaF, 1 mM Na_3_VO_4_ and 1 mM PMSF) containing protease inhibitor cocktail. The mixture was kept on ice for 15 min. After centrifugation, the supernatant was used as nuclear lysates. Protein concentration in the supernatant was determined by the Bio-Rad protein assay. Equal quantities of protein were subjected to SDS-PAGE and transferred to Immobilon-P membrane. The membranes were blocked and then incubated with the primary antibody overnight at 4 °C followed by an appropriate secondary antibody. Secondary antibody binding was visualized using a Chemi-Lumi One Super. Densitometric analysis of the bands was carried out using the Image J Software Program (National Institutes of Health, Bethesda, MD, USA).

### NF-*κ*B luciferase assay

Cells were cultured in 24-well plates (5 × 10^4^ cells/well) in normal growth medium and cotransfected with 1 *μ*g of pNF-*κ*B-Luc and 1 *μ*g of pRL-TK vector (internal control) for 48 h using Lipofectamine 3000 and treated with BITC for 6 h. After treatment, cells were lysed and analyzed using a Dual-Luciferase Reporter Assay System.

### RT-PCR

Cells were washed with ice-cold PBS (-). Total cellular RNA was isolated using Trizol reagent according to the manufacturer's recommendations. RNA was quantified by measuring absorbance at 260 nm. Total RNA (8 *μ*g) was reverse transcribed with Oligo dT to cDNA using M-MLV reverse transcriptase. PCR amplification was then performed with Taq polymerase and specific primers. Primers used in PCR amplification are as follows: cyclin D1, 5′-TCAAGTGTGACCGAGACTGC-3′ and 5′-AGAGATGGAAGGGGGAAAGA-3′ (355 bp); *β*-actin, 5′-GTCACCCACACTGTGCCCATCTA-3′ and 5′-GCAATGCCAGGGTACATGGTGGT-3′ (455 bp). The PCR products were then subjected to agarose gel electrophoresis (3%), stained with ethidium bromide and photographed. Densitometric analysis of the bands was carried out using the Image J Software Program.

### MTT assay

MTT assay was carried out for quantitative analysis of cell viability according to the manufacturer's instructions. Cells were pre-incubated for 24 h in 96-well plate and treated with BITC for 24 h at 37 °C. MTT solution was added to each well, and the absorbance was measured at 570 nm after 2-h incubation at 37 °C. The obtained values were compared with each of the controls incubated with vehicle only.

### Immunoprecipitation assay

Whole-cell lysates (800 *μ*g), prepared as described above, were used for immunoprecipitation for 1 h at 4 °C with *β*-catenin antibody, p65 antibody or goat IgG. Following immunoprecipitation, 20 *μ*l Protein A/G PLUS-Agarose Immunoprecipitation reagents were added and the mixture was incubated for 2 h at 4 °C. Beads were washed with lysis buffer three times. Immunoprecipitated proteins were subjected to Western blot analysis.

### Chromatin immunoprecipitation (ChIP) assay

Cells were cross-linked with 1% formaldehyde at 37 °C. Cells were washed with PBS (-) containing 1 mM PMSF and 1 mM protease inhibitor cocktail, lysed with SDS lysis buffer (50 mM Tris-HCl, 10 mM EDTA, 1% SDS, 1 mM PMSF and 1 mM protease inhibitor cocktail, pH 8.1) and sheared by sonication. The lysates were centrifuged and the supernatants were collected. Soluble chromatin was precleared with salmon sperm DNA and Protein A/G PLUS-Agarose Immunoprecipitation reagent for 30 min and immunoprecipitated with *β*-catenin antibody, p65 antibody or goat IgG overnight at 4 °C. Control sample was omitted the inclusion of antibody. Immune complexes were collected with Protein A/G PLUS-Agarose beads for 2 h and washed once with low-salt buffer (0.1% SDS, 1% Triton X-100, 2 mM EDTA, 20 mM Tris-HCl and 150 mM NaCl, pH 8.1), once with high-salt buffer (0.1% SDS, 1% Triton X-100, 2 mM EDTA, 20 mM Tris-HCl and 500 mM NaCl, pH 8.1), once with LiCl buffer (0.25 M LiCl, 1% NP-40, 1% deoxycholate, 1 mM EDTA and 10 mM Tris-HCl, pH 8.1) and twice with TE buffer and extracted with elution buffer (1% SDS, 0.1 M NaHCO_3_ and 1 mM DTT). The eluent was added with 200 mM NaCl and heated at 65 °C for 6 h to reverse the formaldehyde cross-linking. After digestion with RNase and proteinase K, DNA fragments were purified by phenol extraction and ethanol precipitation. Purified DNA fragments were used as a template for PCR amplification. The PCR products were then subjected to agarose gel electrophoresis (5%), stained with ethidium bromide and photographed. Densitometric analysis of the bands was carried out using the Image J Software Program. Primers used for ChIP assay are as follows and referred to the previous report^43^: TBE0 site on cyclin D1 promoter (−551 to −433), 5′-GGTCCTCCCCGTCCTTGC-3′ and 5′-TGGCGTTCTTGGAAATGCG-3′ and NF-*κ*B binding site on cyclin D1 promoter (−872 to −782), 5′-GCTTTCCATTCAGAGGTGTGTT-3′ and 5′-GTCAAGGTAGGAAGGCAGCC-3′.

## Abbreviations

BITC: benzyl isothiocyanate
NF-*κ*B: nuclear factor-*κ*B
APC: adenomatous polyposis coli
TCF: T-cell factor
IKK: I*κ*B kinase
ITCs: isothiocyanates
LDH: lactate dehydrogenase
SFN: sulforaphane
FBS: fatal bovine serum
TBE0: TCF-binding element0
*O*-GlcNAc: *O*-linked *β*-*N*-acetyl glucosamine
