# Supplementary material for: Nuclear factor-kappaB sensitizes to benzyl isothiocyanate-induced antiproliferation in p53-deficient colorectal cancer cells
Source: Cell Death Dis. 2014 Nov 20;5(11):e1534–. doi: 10.1038/cddis.2014.495 (PMC4260753; doi:10.1038/cddis.2014.495)
Supplement: Supplementary Information [file cddis2014495x1.doc]

**Supplementary information**

Nuclear factor-kappaB sensitizes to benzyl isothiocyanate-induced anti-proliferation in p53-deficient colorectal cancer cells*

*Running title: *NF-κB sensitizes to anti-proliferation by BITC*

**Naomi Abe^1, 2^, De-Xing Hou^3^, Shintaro Munemasa^1^, Yoshiyuki Murata^1^ and Yoshimasa Nakamura^1^**

^1^Graduate School of Environmental and Life Science, Okayama University, Okayama 700-8530, Japan

^2^Research Fellow of Japan Society for the Promotion of Science

^3^Department of Biochemical Science and Technology, Faculty of Agriculture, Kagoshima University, Korimoto 890-8580, Japan

To whom correspondence should be addressed: Yoshimasa Nakamura, Graduate School of Environmental and Life Science, Okayama University, Okayama 700-8530, Japan, Tel&Fax: +81-86-251-8300; E-mail: yossan@cc.okayama-u.ac.jp

**Materials and Methods**

**Chemicals and antibodies.** Benzyl isothiocyanate (BITC) was purchased from LKT Laboratories, Inc. (St Paul, MN). Antibodies against phosphorylated IκB-α (phospho-IκB-α, Ser32/36) was purchased from Cell Signaling Technology, Inc. (Beverly, MA). antibodies against IκB-α and actin, and horseradish peroxidase-linked anti-rabbit and anti-mouse IgGs were purchased from Santa Cruz Biotechnology (Santa Cruz, CA). Protease inhibitor cocktail was purchased from Sigma-Aldrich (St. Louis, MO). McCoy’s 5A medium, penicillin/streptomycin and Trizol reagent were purchased from Life technologies (Carlsbad, CA). Fatal bovine serum (FBS) was purchased from Nichirei Corporation (Tokyo, Japan). Bio-Rad Protein Assay was purchased from Bio-Rad Laboratories (Hercules, CA). Chemi-Lumi One Super was purchased from Nakalai Tesque Inc. (Kyoto, Japan). Immobilon-P membrane was purchased from Merck Millipore (Billerica, MA). M-MLV reverse transcriptase and Taq polymerase were purchased from Takara Bio Inc. (Shiga, Japan). All other chemicals were purchased from Wako Pure Chemical Industries (Osaka, Japan).

**Human colorectal cancer cell lines.** HT-29 cells and HCT-116 p53^+/+^ cells were obtained from the American Type Culture collection (ATCC) (Manassas, VA). Cells were maintained in McCoy’s 5A medium supplemented with 10% heat-inactivated FBS and 1% penicillin/streptomycin. Cells were grown at 37°C in an atmosphere of 95% and 5% CO_2_. Confluent cells were exposed to the test compounds (resolved in 0.2% DMSO) in the medium containing 0.5% FBS.

**Western blot analysis.** Cells were washed with ice-cold PBS (-). Whole cell lysates were prepared in lysis buffer (20 mM Tris-HCl pH 7.5, 150 mM NaCl, 2 mM EDTA, 2 mM EGTA, 2 mM DTT, 10 mM NaF, 1 mM Na_3_VO_4_, 1 mM PMSF, 1% SDS and 1% Triton-X100) containing protease inhibitor cocktail and left on ice for 20 min. After sonication, lysates were centrifuged and the supernatant was used as whole cell lysates. Protein concentration in the supernatant was determined by the Bio-Rad protein assay. Equal quantities of protein were subjected to SDS-PAGE and transferred to Immobilon-P membrane. The membranes were blocked and then incubated with the primary antibody overnight at 4°C followed by an appropriate secondary antibody. Secondary antibody binding was visualized using a Chemi-Lumi One Super. Densitometric analysis of the bands was carried out using the Image J Software Program.

**RT-PCR.** Cells were washed with ice-cold phosphate buffered saline (PBS) (-). Total cellular RNA was isolated using Trizol reagent according to the manufacturer’s recommendations. RNA was quantified by measuring absorbance at 260 nm. Total RNA (8 μg) was reverse transcribed with Oligo dT to cDNA using M-MLV reverse transcriptase. PCR amplification was then performed with Taq polymerase and specific primers. Primers used in PCR ampliﬁcation are as follows: c-myc, 5’-GTCCTCGGATTCTCTGCTC-3’ and 5’-GACTCTGACACTGTCCAACT-3’ (342 bp); COX-2, 5’-TTCAAATGAGATTGTGGAAAAATTGCT-3’ and 5’-AGATCATCTCTGCCTGAGTATCTT-3’ (305 bp); and interferon-γ (IFN-γ), 5’-GGTCATTCAGATGTAGCGGA-3’ and 5’-GCGTTGGACATTCAAGTCAG-3’ (270 bp); β-actin, 5’-GTCACCCACACTGTGCCCATCTA-3’ and 5’-GCAATGCCAGGGTACATGGTGGT-3’ (455 bp). The PCR products were then subjected to agarose gel electrophoresis (3%), stained with ethidium bromide and photographed. Densitometric analysis of the bands was carried out using the Image J Software Program.

**Figure legends for supplementary Figure**

**Figure 1** Effects of BITC on the mRNA level of c-myc, COX-2, IFN-γ. HT-29 cells were treated with BITC for 6 h. The mRNA levels of c-myc, COX-2, IFN-γ and β-actin were determined by RT-PCR.

**Figure 2** Effects of BITC on the phosphorylation of IκB-α in HCT-116 p53^+/+^ cells. HCT-116 p53^+/+^ cells were treated with the indicated concentrations of BITC for 3 h. Western blot analysis of whole cell lysates was performed for p-IκB-α (Ser32/36),  IκB-α and actin.
