# Supplementary figures and images for: Nuclear factor-kappaB sensitizes to benzyl isothiocyanate-induced antiproliferation in p53-deficient colorectal cancer cells
Source: Cell Death Dis. 2014 Nov 20;5(11):e1534–. doi: 10.1038/cddis.2014.495 (PMC4260753; doi:10.1038/cddis.2014.495)

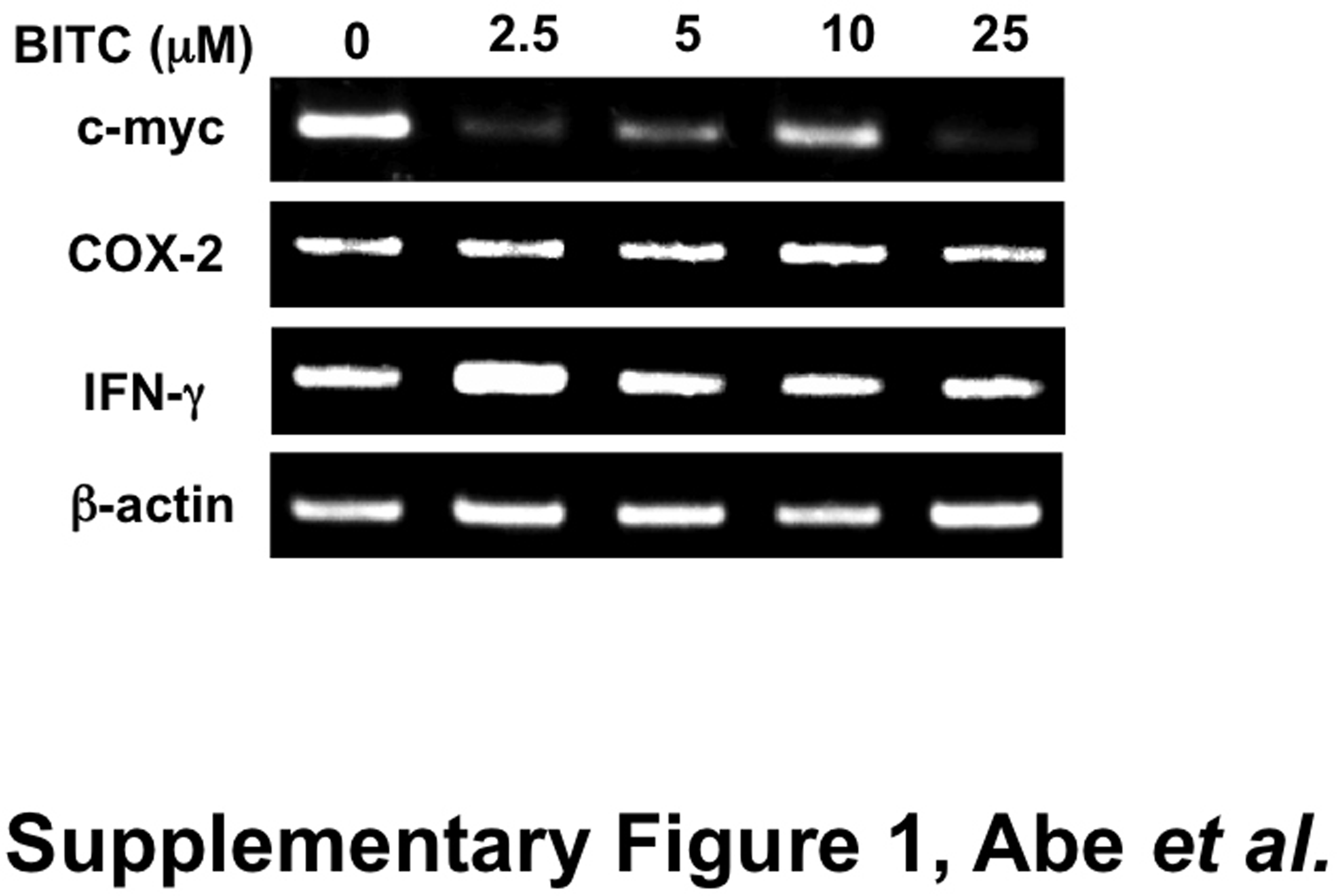

Supplement: Supplementary Figure 1 [file cddis2014495x2.tif]

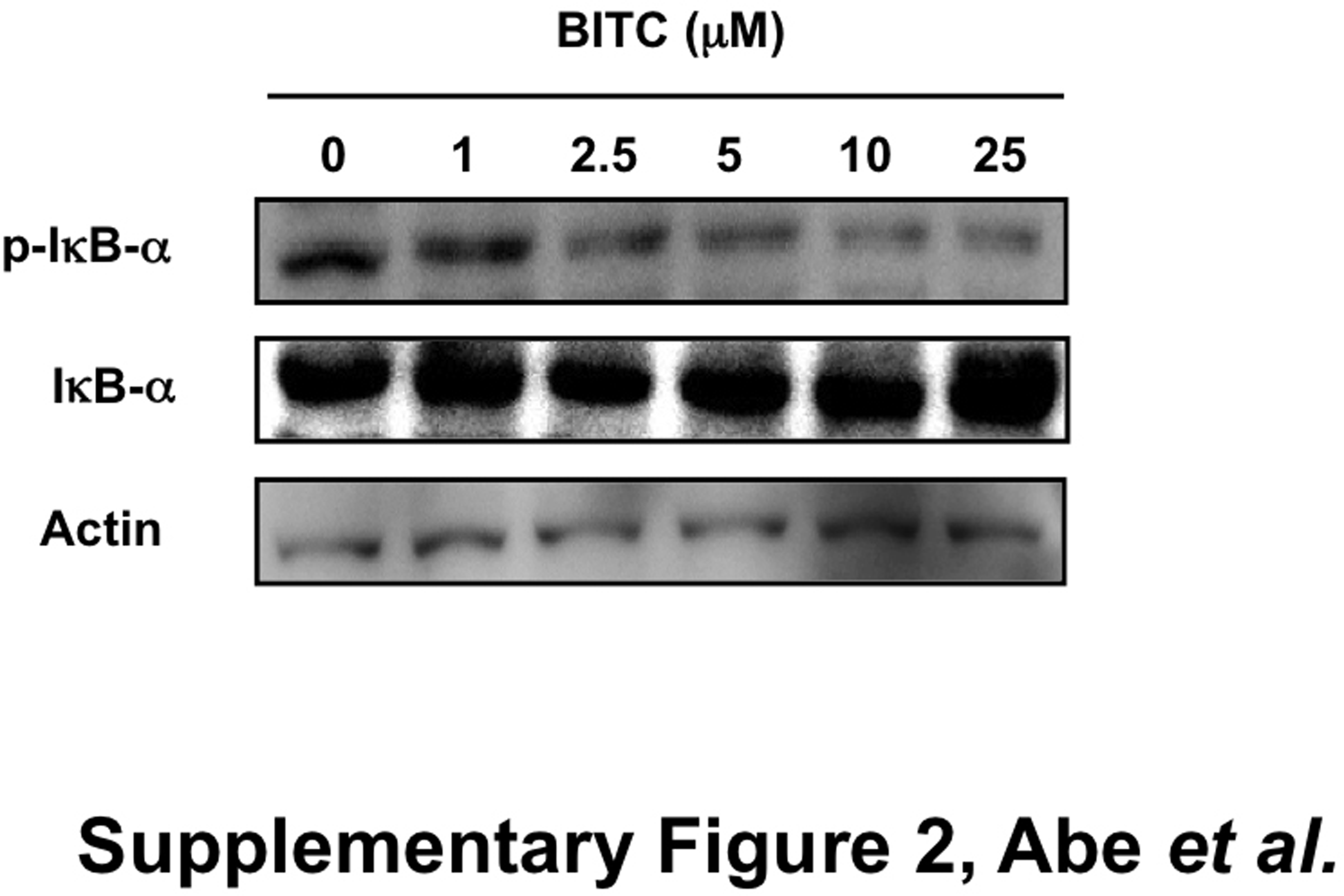

Supplement: Supplementary Figure 2 [file cddis2014495x3.tif]
